# A Fragmentation Study on Four Unusual Secoiridoid Trimers, Swerilactones H–K, by Electrospray Tandem Mass Spectrometry

**DOI:** 10.1007/s13659-016-0114-6

**Published:** 2016-11-14

**Authors:** Chang-An Geng, Ji-Jun Chen

**Affiliations:** 1 State Key Laboratory of Phytochemistry and Plant Resources in West China, Kunming Institute of Botany, Chinese Academy of Sciences, No. 132 Lanhei Road, Kunming, 650201 China; 2 Yunnan Key Laboratory of Natural Medicinal Chemistry, Kunming, 650201 China

**Keywords:** ESI-IT-TOF-MS^*n*^, Fragmentation rules, Secoiridoid trimers, Swerilactones H–K, Retro-Diels–Alder (RDA) cleavage

## Abstract

Swerilactones H–K (**1**–**4**) as four unprecedented secoiridoid trimers represent a new type of natural product, which has attracted much interest of natural chemists due to their novel skeletons and promising bioactivity. In order to well understand their MS fragmentation behaviors, they were investigated by electrospray ionization ion-trap time-of-flight multistage product ion mass spectrometry (ESI-IT-TOF-MS^*n*^) for the first time. The protonated molecules ([M+H]^+^) of swerilactones J and K, and deprotonated molecules ([M−H]^−^) of swerilactones H, J and K were readily observed in the conventional single-stage mass spectra (MS); however only the [M+Cl]^−^ ion for swerilactone I was obtained in negative mode. Based on the MS^*n*^ study, the fragmentation pathways of swerilactones H and I in negative mode, and swerilactones J and K in both positive and negative modes were proposed. The neutral losses of H_2_O, CO, CO_2_ and C_2_H_4_O moieties are the particular elimination from the precursor ions due to the presence of hydroxyl, *δ*-lactone and 1-*O*-ethyl moieties in their structures, of which the retro-Diels–Alder cleavage was the most particular dissociation. The fragment ions at *m/z* 341 and 291 in negative mode can be considered as the diagnostic ions for secoiridoid trimers. This investigation will provide valuable information for their fast characterization from complicated natural mixtures and extensive understanding their structural architectures.

**Electronic supplementary material:**

The online version of this article (doi:10.1007/s13659-016-0114-6) contains supplementary material, which is available to authorized users.

## Introduction

**Fig. 1:**
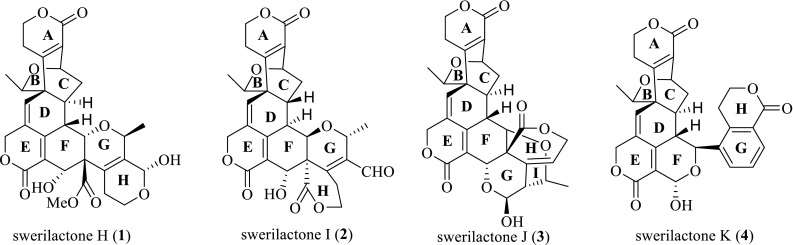
Structures of compounds **1**–**4**

Natural products with diversities in chemical structures and pharmacological activities provide versatile candidates in drug discovery. Many natural chemists are committed to searching for novel compounds to enrich this library. Swerilactones H–K (**1**–**4**) (Fig. 1), unprecedented secoiridoid trimers from the traditional Chinese herb *Swertia mileensis*, represent a new type of natural product, which has attracted much interest of natural chemists due to their novel skeletons and promising bioactivity [1, 2]. However, this type of compound exists as minor components in plants, which presents challenges for their fast and reliable characterization [3]. Mass spectrometry (MS) with the associated high sensitivity and resolution well meets this requirement and has become the routine method in various aspects of medicinal chemistry [4–8]. Tandem MS techniques are particularly useful for ascertaining the relationship between precursor and product ions, by which the fragmentation rules and diagnostic ions of complicated compounds can be easily deduced [9–14]. The LCMS-IT-TOF mass spectrometer equipped with an electrospray ionization source linked to ion-trap and time-of-flight mass analyzers (ESI-IT-TOF) allows fast acquisition of multistage product ion spectra (MS^*n*^) with high accuracy and resolution in both positive and negative modes [15–17]. This feature leads to easier interpretation of the origin of product ions, which is suitable for investigating the structures of natural products. In this paper, we report for the first time a high-resolution MS^*n*^ fragmentation study on swerilactones H–K (**1**–**4**) by ESI-IT-TOF mass spectrometer, which will provide valuable information not only for their fast characterization from complicated natural mixtures but also for a better understanding of their structural architectures.

## Experimental

### Apparatus and Analytical Conditions

MS^*n*^ analyses were acquired on the LCMS-IT-TOF mass spectrometer (Shimadzu, Kyoto, Japan). The mass resolution was about 10000 full width at half maximum (FWHM). Accurate masses were corrected by calibration using sodium trifloroacetate (CF_3_CO_2_Na) clusters. MS experiments were achieved in automatic pattern, and MS^*n*^ experiments were performed in direct mode. Unless specified otherwise, analytical conditions were as follows: spray voltage, 4.50 and −3.50 kV; detector voltage, 1.60 kV; drying gas pressure, 100.0 kPa; nebulizing gas (N_2_) flow, 0.5 L/min; curved desolvation line (CDL) temperature, 200.0 °C; heat block temperature, 200.0 °C; equipment temperature, 40.0 °C; ion accumulation time, 10 ms; precursor ion selected width, *m/z* ± 3.0 Da, and selected time, 20 ms; collision induced dissociation (CID) collision time, 30 ms; collision energy, 50%; collision gas, 50%; and q = 0.251; scan range, *m/z* 100–1000 for MS. The Shimadzu Composition Formula Predictor was used to determine the molecular formula.

### Chemicals and Samples

HPLC grade acetonitrile (CH_3_CN) was purchased from Merck (Merck Co. Ltd., Germany). HPLC grade formic acid was purchased from Aladdin (Aladdin Chemistry Co. Ltd. China). Deionized water was purified using a MingChe™-D 24UV Merck Millipore system (Merck Millipore, Shanghai, China).

Swerilactones H–K (**1**–**4**) were isolated from *S. mileensis* in our previous investigation, whose structures were unambiguously determined by extensive spectroscopic data and X-ray analyses [1]. Sample solutions were prepared by dissolving each sample in a solution of 85% CH_3_CN/H_2_O containing 0.05% formic acid to a final concentration of 0.2 mg/mL. The samples were introduced into the source via a syringe pump at a flow rate of 2 μL/min.

## Results and Discussion

**Fig. 2:**
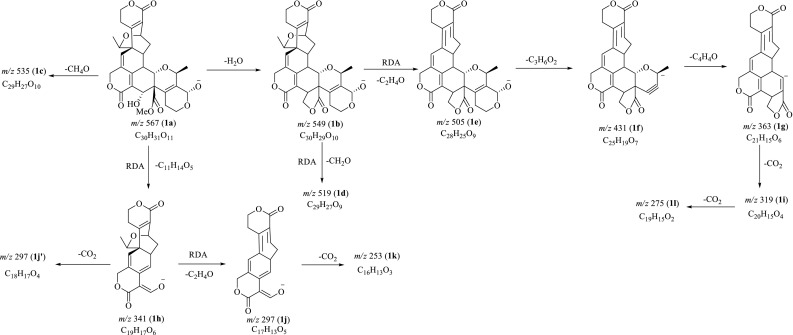
Proposed fragmentation pathways of swerilactone H (**1**) in negative mode

**Fig. 3:**
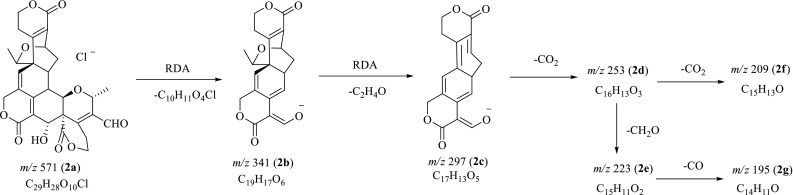
Proposed fragmentation pathways of swerilactone I (**2**) in negative mode

**Fig. 4:**
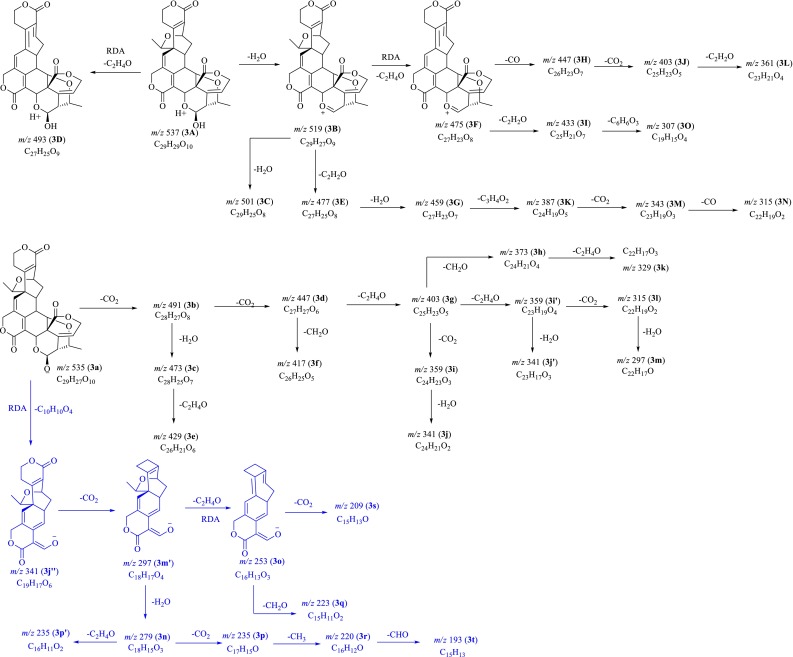
Proposed fragmentation pathways of swerilactone J (**3**) in positive and negative modes

**Fig. 5:**
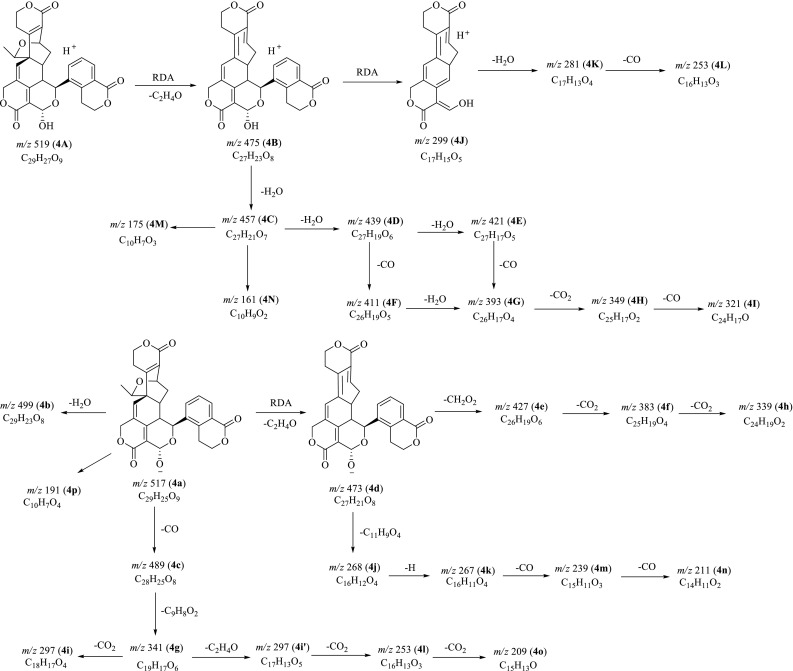
Proposed fragmentation pathways of swerilactone K (**4**) in positive and negative modes

Before MS^*n*^ investigation, the full-scan MS of compounds **1**–**4** in both positive and negative ion modes were acquired in automatic pattern. The protonated molecule ([M+H]^+^) and deprotonated molecule ([M–H]^−^) ions for swerilactones J (**3**) and K (**4**) were readily detected. However, swerilactones H (**1**) and I (**2**) only displayed [M–H]^−^ or [M+Cl]^−^ ion in negative mode. Therefore, the subsequent MS^*n*^ study for swerilactones H and I (**1** and **2**) in negative mode, and for swerilactones J and K (**3** and **4**) in both positive and negative modes was performed, from which their fragmentation pathways were proposed (Figs. 2, 3, 4, 5). It should be noted that alternative ways of fragmentation that can reasonably interpret the product ions are also possible in addition to the proposed pathway.

### ESI-IT-TOF MS^*n*^ Fragmentations of Swerilactone H (**1**) in Negative Mode

In the single-stage mass spectrum of swerilactone H (**1**), the deprotonated molecular [M–H]^−^ ion at *m/z* 567.1871 (**1a**) was readily obtained, corresponding to the molecular formula C_30_H_32_O_11_. When [M–H]^−^ (**1a**) was selected as the precursor ion to perform MS^2^ experiment, multiple product ions (**1b**–**1k**) were observed. Among them, the ions at *m/z* 549 (**1b**) and 535 (**1c**) were deduced to be generated by the neutral losses of H_2_O and CH_4_O from **1a** due to the presence of vicinal hydroxyl and methoxy groups [18, 19]. The product ions at *m/z* 519 (**1d**) and 505 (**1e**) were assigned to be the elimination of CH_2_O and the retro-Diels–Alder (RDA) cleavage of C_2_H_4_O from **1b** [20]. Similarly, the cleavage of ring H by losing one C_3_H_6_O_2_ fragment from **1e** generated ion **1f** at *m/z* 431, and the most abundant ion at *m/z* 363 (**1g**) could be explained by subsequent loss of a C_4_H_4_O molecule by an RDA-like process [21]. It is noteworthy that two abundant ions at *m/z* 341 (**1h**) and 297 (**1j**/**1j′**) were readily observed in the MS^2^ spectrum, of which the ions at *m/z* 297 were present as double peaks at *m/z* 297.0792 (**1j**) and 297.1105 (**1j′**), corresponding to the chemical composition of C_17_H_13_O_5_ and C_18_H_17_O_4_ (Fig. 2). The ion at *m/z* 341 (**1h**) could be well interpreted by the RDA cleavage of ring F to lose a C_11_H_14_O_5_ part [22, 23]. Thus, the elimination of 44 Da can be attributed to the losses of C_2_H_4_O and CO_2_ to yield product ions **1j** (*m/z* 297.0792) and **1j′** (*m/z* 297.1105) [24]. Likewise, the ion **1j** could further lose a molecule of CO_2_ to generate ion **1k** (*m/z* 253). In the MS^3^ experiment from the precursor ion **1g** (*m/z* 363), two product ions at *m/z* 319 (**1i**) as base peak and 275 (**1l**) were detected, which were proposed to be arisen from the sequential loss of two CO_2_ molecules.

### ESI-IT-TOF MS^*n*^ Fragmentations of Swerilactone I (**2**) in Negative Mode

Compared to swerilactones H, J and K, swerilactone I (**2**) was more unstable in this MS^*n*^ study, and thus, gave rise to less MS^*n*^ information, which might be due to the presence of aldehyde group in the structure. In the full-scan mass spectrum, swrilactone I (**2**) displayed neither [M+H]^+^ nor [M−H]^−^ ions, but an ion at *m/z* 571.1383 (**2a**) was readily obtained in the negative ion mode. This ion was ascribed with the chemical composition of C_29_H_28_O_10_Cl ([M+Cl]^−^) based on its high accordance in both accuracy (0.7 mDa) and isotopic abundance (83.9%) with those of the theoretical values. However, the origin of Cl^−^ ion was unclear, which was always encountered in negative ESIMS investigation [25, 26]. In addition to the [M+Cl]^−^ ion, two fragments at *m/z* 341 (**2b**) and 297 (**2c**) were observed with high abundance. The ion **2b** corresponding to the loss of a C_10_H_11_O_4_Cl motif (rings G and H) from ion **2a** could be explained by the RDA ring-opening of ring F, and the ion **2c** was proposed to be generated by a further RDA process leading to the loss of a C_2_H_4_O part from ion **2b** (Fig. 3). The above deduction was confirmed by the MS^2^ experiment on **2a**, which gave rise to the expected ions **2b** (*m/z* 341) and **2c** (*m/z* 297), and the subsequent MS^3^ experiment on **2b** in which the fragment ion at *m/z* 297 (**2c**) was further detected. Combined with the observation that the fragmentation ion **2c** showed much higher abundance compared to the parent [M+Cl]^−^ ion in the first stage mass spectrum, the following MS^2^ experiment was further performed on ion **2c** (*m/z* 297) to generate three characteristic ions at *m/z* 253 (**2d**), 223 (**2e**) and 209 (**2f**). The ion **2d** was attributed to the neutral loss of a CO_2_ moiety from ion **2c**, and the ions **2e** and **2f** were corresponding to the elimination of one CH_2_O or CO_2_ parts from **2d**. In the MS^3^ experiment on ion **2d**, the expected fragment ions **2e** (*m/z* 223) and **2f** (*m/z* 209) were readily displayed, which was in accordance with the above deduction. When ion **2e** was further selected for MS^4^ experiment, a fragment ion at *m/z* 195 (**2g**) corresponding to a 28 Da loss was obtained, which was deduced as the elimination of one CO moiety from the ion **2e** [18].

### ESI-IT-TOF MS^*n*^ Fragmentations of Swerilactone J (**3**) in Positive and Negative Modes

In the positive full-scan mass spectrum, the [M+H]^+^ ion (**3A**) at *m/z* 537.1732 was readily detected, as well as the fragment ion (**3B**) at *m/z* 519 ([M+H–H_2_O]^+^) which was displayed as base peak in the subsequent MS^2^ experiment from **3A**. The MS^2^ product ion at *m/z* 493 (**3D**) was designated as the RDA elimination of C_2_H_4_O moiety from **3A** due to the presence of 1-O-ethyl group in the structure (Fig. 4). Similarly, the ion **3F** (*m/z* 475) was formed by losing a C_2_H_4_O part from **3B**, and further gave rise to ions **3H** (*m/z* 447) and **3J** (*m/z* 403) via consecutive elimination of one CO and one CO_2_ molecule [20]. The loss of a C_2_H_2_O segment was characteristic, by which the fragments **3E** (*m/z* 477), **3I** (*m/z* 433) and **3L** (*m/z* 361) were formed from their respective parent ions **3B**, **3F** and **3J**. In the MS^3^ spectrum from **3B**, the ion **3C** (*m/z* 501) corresponding to the loss of a H_2_O molecule was observed. With the elimination of a C_3_H_4_O_2_ part, the ion **3K** (*m/z* 387) was produced, and further generated ions **3M** (*m/z* 343) and **3N** (*m/z* 315) by successive losses of CO_2_ and CO molecules. The ion **3O** (*m/z* 307) in the MS^3^ spectrum was correspondent to the elimination of C_6_H_6_O_3_ moiety from precursor **3I**.

The MS^*n*^ investigation on swerilactone J (**3**) in negative mode provided more valuable information than that in positive mode. The first-stage mass spectrum displayed the [M–H]^−^ ion at *m/z* 535.1602, assigned to the molecular formula C_29_H_28_O_10_. It should be noted that two fragmentation ions at *m/z* 491 (**3b**) and *m/z* 341 (**3j″**) were readily obtained with high abundance in addition to the [M–H]^−^ ion, assigned to the molecular formula C_28_H_27_O_8_ and C_19_H_17_O_6_, respectively. The ion **3b** was explained by the neutral loss of CO_2_ from the precursor ion **3a**, and further confirmed by MS^2^ analysis in which the ion at *m/z* 491 was obtained as base peak. The ion **3j″** (C_19_H_17_O_6_) was proposed to be derived from **3a** by neutral loss of a C_10_H_10_O_4_ part, due to the RDA cleavage of ring F [20]. When ion **3b** (*m/z* 491) was selected as the precursor ion to perform MS^2^ experiment, prolific fragment ions were obtained, from which their fragmentation rules were proposed as shown in Fig. 4. Due to the high abundance of ion **3j″** (*m/z* 341.1030, C_19_H_17_O_6_) in the first-stage mass spectrum, subsequent MS^2–4^ experiments were applied on ion **3j″**, from which a parallel fragmentation pathway was recognized. The neutral loss of CO_2_ from **3j″** provided ion **3** **m′** (*m/z* 297.1081), and further produced ions **3n** (*m/z* 279) and **3o** (*m/z* 253) through the elimination of H_2_O or C_2_H_4_O part. In the MS^4^ experiment from the precursor ion **3n** (*m/z* 279), characteristic product ions at *m/z* 235, 220 and 193 were obtained, of which the ion at *m/z* 235 was consist of two closed peaks at *m/z* 235.1087 (C_17_H_15_O, **3p**) and 235.0749 (C_16_H_11_O_2_, **3p′**), attributed to the neutral losses of CO_2_ or C_2_H_4_O moiety from **3n**.

### ESI-IT-TOF MS^*n*^ Fragmentations of Swerilactone K (**4**) in Positive and Negative Modes

Structurally, swerilactone K (**4**) with an aromatic ring is obviously different from swerilactones H–J (**1**–**3**). The first-stage mass spectrum in positive mode displayed [M+H]^+^ ion (**4A**) at *m/z* 519.1651, corresponding to the molecular formula C_29_H_26_O_9_. The subsequent MS^2^ experiment from **4A** yielded two high-abundance ions **4B** (*m/z* 475) and **4C** (*m/z* 457, base peak), attributed to the successive losses of C_2_H_4_O and H_2_O parts, in combination with three minor ions at *m/z* 299 (**4J**), 281(**4k**) and 253 (**4L**). The ion **4J** was interpreted by the neutral loss of C_10_H_8_O_3_ from the precursor **4B** due to the RDA cleavage of ring F, and further gave rise to ions **4K** (*m/z* 281) and **4L** (*m/z* 253) by the elimination of a molecule of H_2_O and CO (Fig. 5). This deduction was also confirmed by the MS^3^ analysis from the parent ion **4B**. When ion **4C** (*m/z* 457) was applied for the MS^4^ experiment, the most intensive ion at *m/z* 439 (**4D**) was readily detected, ascribe to the loss of H_2_O, together with a series of fragment ions **4E**−**4I**.

In the negative ion mode, sweilactone K (**4**) gives rise to the deprotonated ion at *m/z* 517.1511, correlated to the molecular formula C_29_H_26_O_9_. The following MS^2^ experiment on **4a** provided versatile fragments with ion at *m/z* 473 (**4d**) as base peak which was further applied for MS^3^ spectrum. Based on the above experiments, the fragmentation rules for swerilactone K (**4**) in negative mode were concluded. The minor ions at *m/z* 499 (**4b**) and 489 (**4c**) in MS^2^ spectrum were derived from neutral loss of H_2_O and CO from the precursor **4a**. The most abundant ion **4d** (*m/z* 473) generated from **4a** by the RDA elimination of C_2_H_4_O segment, can further give rise to ions at *m/z* 427 (**4e**), 383 (**3f**) and 339 (**4h**) by sequential losses of CH_2_O_2_, CO_2_ and CO_2_ parts. In the MS^3^ spectrum from **4d**, the product ions **4j** (*m/z* 268), **4k** (*m/z* 267), **4m** (*m/z* 239) and **4n** (*m/z* 211) could be explained by the consecutive elimination of C_11_H_9_O_4_ radical, hydrogen radical, CO and CO, respectively.

## Conclusion

The ESI multistage product ion mass spectra (MS^*n*^) of swerilactones H–K were obtained for the first time by LCMS-IT-TOF, from which their fragmentation pathways were deduced. This investigation suggested that these molecules were unstable in this MS^*n*^ study, especially for swerilactone I. The losses of H_2_O, CO_2_, CO and C_2_H_4_O moieties were the particular elimination from the precursor ions due to the presence of hydroxyl, *δ*-lactone and 1-*O*-ethyl groups. In particular, the RDA dissociation was the most common fragmentation rule which might correspond to the fused six-membered rings in their structures. It is important to note that the loss of CO_2_ and C_2_H_4_O can be unambiguously distinguished by high-resolution mass spectrometry. Structurally, swerilactones H–K share a closely related skeleton with the main difference located at rings F, G and H. Therefore, the conservative moiety (rings A to E) leads to the common fragments at *m/z* 341 and 291 in negative mode, which can be considered as the diagnostic ions for secoiridoid trimers. The present MS^*n*^ fragmentation study on swerilactones H–K (**1**–**4**) by ESI-IT-TOF mass spectrometer will provide valuable information not only for their fast characterization from complicated natural mixtures but also for a better understanding of their structural architectures.

## Electronic supplementary material

Below is the link to the electronic supplementary material.

Supplementary material 1 (DOCX 357 kb)
