# Supplementary material for: A Fragmentation Study on Four Unusual Secoiridoid Trimers, Swerilactones H–K, by Electrospray Tandem Mass Spectrometry
Source: Nat Prod Bioprospect. 2016 Nov 14;6(6):297–303. doi: 10.1007/s13659-016-0114-6 (PMC5136375; doi:10.1007/s13659-016-0114-6)
Supplement: Supplementary file 1 — Supplementary material 1 (DOCX 357 kb) [file 13659_2016_114_MOESM1_ESM.docx]

Figure S1. Mass spectra of swerilactone H (**1**) in negative mode

Figure S2. Mass spectra of swerilactone I (**2**) in negative mode

Figure S3. Mass spectra of swerilactone J (**3**) in positive mode

Figure S4. Mass spectra of swerilactone J (**3**) in negative mode

MS (–)

Figure S5. Mass spectra of swerilactone K (**4**) in positive mode

MS(+)

Figure S6. Mass spectra of swerilactone K (**4**) in negative mode

MS(–)

**Table 1** Data for accurate masses and elemental composition of swerilactone H (**1**) observed from tandem mass spectra in negative mode.

| MS^n^ | Precursor  ion (*m/z*) | Product  ion (*m/z*) | Elemental  composition | Measured  (*m/z*) | Calculated  (*m/z*) | Error  (mDa) | Ion  name | Assignment |
| --- | --- | --- | --- | --- | --- | --- | --- | --- |
| (–) MS | (568) | 567 | C_30_H_31_O_11_ | 567.1871 | 567.1872 | –0.1 | **1a** | [M–H]^–^ |
| MS^2^ | 567 | 549 | C_30_H_29_O_10_ | 549.1774 | 549.1766 | +0.8 | **1b** | **1a**–H_2_O |
|  |  | 535 | C_29_H_27_O_10_ | 535.1587 | 535.1610 | –2.3 | **1c** | **1a**–CH_3_OH |
|  |  | 519 | C_29_H_27_O_9_ | 519.1687 | 519.1661 | +2.6 | **1d** | **1b**–CH_2_O |
|  |  | 505 | C_28_H_25_O_9_ | 505.1475 | 505.1505 | –3.0 | **1e** | **1b**–C_2_H_4_O |
|  |  | 431 | C_25_H_19_O_7_ | 431.1127 | 431.1136 | –0.9 | **1f** | **1e**–C_3_H_6_O_2_ |
|  |  | 363 | C_21_H_15_O_6_ | 363.0858 | 363.0874 | –1.6 | **1g** | **1f**–C_4_H_4_O |
|  |  | 341 | C_19_H_17_O_6_ | 341.1032 | 341.1031 | +0.1 | **1h** | **1a**–C_11_H_14_O_5_ |
|  |  | 319 | C_20_H_15_O_4_ | 319.0984 | 319.0976 | +0.8 | **1i** | **1g**–CO_2_ |
|  |  | 297 | C_17_H_13_O_5_ | 297.0792 | 297.0768 | +2.4 | **1j** | **1h**–C_2_H_4_O |
|  |  | 297 | C_18_H_17_O_4_ | 297.1105 | 297.1132 | –2.7 | **1j′** | **1h**–CO_2_ |
|  |  | 253 | C_16_H_13_O_3_ | 253.0851 | 253.0870 | –1.9 | **1k** | **1j**–CO_2_ |
| MS^3^ | 363 | 319 | C_20_H_15_O_4_ | 319.1007 | 319.0976 | +3.1 | **1i** | **1g**–CO_2_ |
|  |  | 275 | C_19_H_15_O_2_ | 275.1068 | 275.1078 | –1.0 | **1l** | **1i**– CO_2_ |

**Table 2** Data for accurate masses and elemental composition of swerilactone I (**2**) observed from tandem mass spectra in negative mode.

| MS^n^ | Precursor  ion (*m/z*) | Product  ion (*m/z*) | Elemental  composition | Measured  (*m/z*) | Calculated  (*m/z*) | Error  (mDa) | Ion  name | Assignment |
| --- | --- | --- | --- | --- | --- | --- | --- | --- |
| (−) MS | (536) | 571 | C_29_H_28_O_10_Cl | 571.1383 | 571.1376 | +0.7 | **2a** | [M+Cl]^−^ |
|  |  | 341 | C_19_H_17_O_6_ | 341.1028 | 341.1031 | −0.3 | **2b** | **2a**−Cl−C_10_H_11_O_4_ |
|  |  | 297 | C_17_H_13_O_5_ | 297.0756 | 297.0768 | −1.2 | **2c** | **2b**−C_2_H_4_O |
| MS^2^ | 571 | 341 | C_19_H_17_O_6_ | 341.1016 | 341.1031 | −1.5 | **2b** | **2a**−Cl−C_10_H_11_O_4_ |
|  |  | 297 | C_17_H_13_O_5_ | 297.0748 | 297.0768 | −2.0 | **2c** | **2b**−C_2_H_4_O |
| MS^3^ | 341 | 297 | C_17_H_13_O_5_ | 297.0755 | 297.0768 | −1.3 | **2c** | **2b**−C_2_H_4_O |
|  |  | 253 | C_16_H_13_O_3_ | 253.0870 | 253.0870 | 0.0 | **2d** | **2c**−CO_2_ |
|  |  | 209 | C_15_H_13_O | 209.0980 | 209.0972 | +0.8 | **2f** | **2d**−CO_2_ |
| MS^2^ | 297 | 253 | C_16_H_13_O_3_ | 253.0873 | 253.0870 | +0.3 | **2d** | **2c**−CO_2_ |
|  |  | 223 | C_15_H_11_O_2_ | 223.0765 | 223.0765 | 0.0 | **2e** | **2d**−CH_2_O |
|  |  | 209 | C_15_H_13_O | 209.0961 | 209.0972 | −1.1 | **2f** | **2d**−CO_2_ |
| MS^3^ | 253 | 223 | C_15_H_11_O_2_ | 223.0756 | 223.0765 | −0.9 | **2e** | **2d**−CH_2_O |
|  |  | 209 | C_15_H_13_O | 209.0980 | 209.0972 | +0.8 | **2f** | **2d**−CO_2_ |
| MS^4^ | 223 | 195 | C_14_H_11_O | 195.0802 | 195.0815 | −1.3 | **2g** | **2e**−CO |

**Table 3** Data for accurate masses and elemental composition of swerilactone J (**3**) observed from tandem mass spectra in positive and negative modes.

| MS^n^ | Precursor  ion (*m/z*) | Product  ion (*m/z*) | Elemental  composition | Measured  (*m/z*) | Calculated  (*m/z*) | Error  (mDa) | Ion  name | Assignment |
| --- | --- | --- | --- | --- | --- | --- | --- | --- |
| (+) MS | (536) | 537 | C_29_H_29_O_10_ | 537.1732 | 537.1755 | –2.3 | **3A** | [M+H]^+^ |
|  |  | 519 | C_29_H_27_O_9_ | 519.1654 | 519.1650 | +0.4 | **3B** | **3A**–H_2_O |
| MS^2^ | 537 | 519 | C_29_H_27_O_9_ | 519.1659 | 519.1650 | +0.9 | **3B** | **3A**–H_2_O |
|  |  | 493 | C_27_H_25_O_9_ | 493.1538 | 493.1493 | +4.5 | **3D** | **3A**–C_2_H_4_O |
|  |  | 477 | C_27_H_25_O_8_ | 477.1530 | 477.1544 | –1.4 | **3E** | **3B**–C_2_H_2_O |
|  |  | 475 | C_27_H_23_O_8_ | 475.1394 | 475.1387 | +0.7 | **3F** | **3B**–C_2_H_4_O |
|  |  | 459 | C_27_H_23_O_7_ | 459.1461 | 459.1438 | +2.3 | **3G** | **3E**–H_2_O |
|  |  | 447 | C_26_H_23_O_7_ | 447.1401 | 447.1438 | –3.7 | **3H** | **3F**–CO |
|  |  | 433 | C_25_H_21_O_7_ | 433.1257 | 433.1282 | −2.5 | **3I** | **3F**–C_2_H_2_O |
|  |  | 403 | C_25_H_23_O_5_ | 403.1548 | 403.1540 | +0.8 | **3J** | **3H**–CO_2_ |
|  |  | 387 | C_24_H_19_O_5_ | 387.1200 | 387.1227 | −2.7 | **3K** | **3G**–C_3_H_4_O_2_ |
|  |  | 361 | C_23_H_21_O_4_ | 361.1461 | 361.1434 | +2.7 | **3L** | **3J**–C_2_H_2_O |
|  |  | 315 | C_22_H_19_O_2_ | 315.1392 | 315.1380 | +1.2 | **3N** | **3M**–CO |
| MS^3^ | 519 | 501 | C_29_H_25_O_8_ | 501.1526 | 501.1544 | –1.8 | **3C** | **3B**–H_2_O |
|  |  | 475 | C_27_H_23_O_8_ | 475.1369 | 475.1387 | –1.8 | **3F** | **3B**–C_2_H_4_O |
|  |  | 459 | C_27_H_23_O_7_ | 459.1436 | 459.1438 | –0.2 | **3G** | **3E**–H_4_O |
|  |  | 433 | C_25_H_21_O_7_ | 433.1269 | 433.1282 | –1.3 | **3I** | **3F**–C_2_H_2_O |
|  |  | 387 | C_24_H_19_O_5_ | 387.1270 | 387.1227 | +4.3 | **3K** | **3G**–C_3_H_4_O_2_ |
|  |  | 343 | C_23_H_19_O_3_ | 343.1318 | 343.1329 | –1.1 | **3M** | **3K**–CO_2_ |
|  |  | 315 | C_22_H_19_O_2_ | 315.1373 | 315.1380 | –0.7 | **3N** | **3M**–CO |
|  |  | 307 | C_19_H_15_O_4_ | 307.0985 | 307.0965 | +2.0 | **3O** | **3I**–C_6_H_6_O_3_ |
| (–) MS | (536) | 535 | C_29_H_27_O_10_ | 535.1602 | 535.1610 | –0.8 | **3a** | [M–H]^–^ |
|  |  | 491 | C_28_H_27_O_8_ | 491.1700 | 491.1706 | −0.6 | **3b** | **3a**−CO_2_ |
|  |  | 341 | C_19_H_17_O_6_ | 341.1030 | 341.1025 | −0.5 | **3j''** | **3a**−C_10_H_11_O_4_ |
| MS^2^ | 535 | 491 | C_28_H_27_O_8_ | 491.1713 | 491.1711 | +0.2 | **3b** | **3a**–CO_2_ |
| MS^2^ | 491 | 473 | C_28_H_25_O_7_ | 473.1655 | 473.1606 | +4.9 | **3c** | **3b**–H_2_O |
|  |  | 447 | C_27_H_27_O_6_ | 447.1760 | 447.1813 | –5.3 | **3d** | **3b**–CO_2_ |
|  |  | 429 | C_26_H_21_O_6_ | 429.1387 | 429.1344 | +4.3 | **3e** | **3c**–C_2_H_4_O |
|  |  | 417 | C_26_H_25_O_5_ | 417.1729 | 417.1707 | +2.2 | **3f** | **3d**–CH_2_O |
|  |  | 403 | C_25_H_23_O_5_ | 403.1563 | 403.1551 | +1.2 | **3g** | **3d**–C_2_H_4_O |
|  |  | 373 | C_24_H_21_O_4_ | 373.1477 | 373.1445 | +3.2 | **3h** | **3g**–CH_2_O |
|  |  | 359 | C_24_H_23_O_3_ | 359.1618 | 359.1653 | –3.5 | **3i** | **3g**–CO_2_ |
|  |  | 359 | C_23_H_19_O_4_ | 359.1304 | 359.1289 | +1.5 | **3i′** | **3g**–C_2_H_4_O |
|  |  | 341 | C_24_H_21_O_2_ | 341.1566 | 341.1547 | +1.9 | **3j** | **3i**–H_2_O |
|  |  | 341 | C_23_H_17_O_3_ | 341.1233 | 341.1183 | +5.0 | **3j′** | **3i′**–H_2_O |
|  |  | 329 | C_22_H_17_O_3_ | 329.1211 | 329.1183 | +2.8 | **3k** | **3h**–C_2_H_4_O |
|  |  | 315 | C_22_H_19_O_2_ | 315.1415 | 315.1391 | +2.4 | **3l** | **3i′**–CO_2_ |
| MS^3^ | 315 | 297 | C_22_H_17_O | 297.1307 | 297.1285 | +2.2 | **3m** | **3l**–H_2_O |
| MS^2^ | 341 | 297 | C_18_H_17_O_4_ | 297.1081 | 297.1132 | –5.1 | **3m′** | **3j′′**–CO_2_ |
|  |  | 279 | C_18_H_15_O_3_ | 279.1062 | 279.1027 | +3.5 | **3n** | **3m′**–H_2_O |
|  |  | 253 | C_16_H_13_O_3_ | 253.0916 | 253.0870 | +4.6 | **3o** | **3m′**–C_2_H_4_O |
|  |  | 235 | C_17_H_15_O | 235.1163 | 235.1128 | +3.5 | **3p** | **3n**–CO_2_ |
|  |  | 209 | C_15_H_13_O | 209.1000 | 209.0972 | +2.8 | **3s** | **3o**–CO_2_ |
|  |  | 193 | C_15_H_13_ | 193.1062 | 193.1023 | +3.9 | **3t** | **3r**–CO+H |
| MS^3^ | 297 | 279 | C_18_H_15_O_3_ | 279.1034 | 279.1027 | +0.7 | **3n** | **3m′**–H_2_O |
|  |  | 253 | C_16_H_13_O_3_ | 253.0836 | 253.0870 | –3.4 | **3o** | **3m′**–C_2_H_4_O |
|  |  | 235 | C_17_H_15_O | 235.1125 | 235.1128 | –0.3 | **3p** | **3n**–CO_2_ |
|  |  | 223 | C_15_H_11_O_2_ | 223.0760 | 223.0765 | –0.5 | **3q** | **3o**–CH_2_O |
|  |  | 209 | C_15_H_13_O | 209.0958 | 209.0972 | –1.4 | **3s** | **3o**–CO_2_ |
|  |  | 193 | C_15_H_13_ | 193.1032 | 193.1023 | +0.9 | **3t** | **3r**–CO+H |
| MS^4^ | 279 | 235 | C_17_H_15_O | 235.1087 | 235.1128 | –4.1 | **3p** | **3n**–CO_2_ |
|  |  | 235 | C_16_H_11_O_2_ | 235.0749 | 235.0765 | –1.6 | **3p′** | **3n**–C_2_H_4_O |
|  |  | 220 | C_16_H_12_O | 220.0923 | 220.0894 | +2.9 | **3r** | **3p**–CH_3_ |
|  |  | 193 | C_15_H_13_ | 193.1031 | 193.1023 | +0.8 | **3t** | **3r**–CO+H |

**Table 4** Data for accurate masses and elemental composition of swerilactone K (**4**) observed from tandem mass spectra in positive and negative modes.

| MS^n^ | Precursor  ion (*m/z*) | Product  ion (*m/z*) | Elemental  composition | Measured  (*m/z*) | Calculated  (*m/z*) | Error  (mDa) | Ion  name | Assignment |
| --- | --- | --- | --- | --- | --- | --- | --- | --- |
| (+) MS | (518) | 519 | C_29_H_27_O_9_ | 519.1651 | 519.1650 | +0.1 | **4A** | [M+H]^+^ |
| MS^2^ | 519 | 475 | C_27_H_23_O_8_ | 475.1394 | 475.1387 | +0.7 | **4B** | **4A**–C_2_H_4_O |
|  |  | 457 | C_27_H_21_O_7_ | 457.1291 | 457.1282 | +0.9 | **4C** | **4B**–H_2_O |
|  |  | 439 | C_27_H_19_O_6_ | 439.1193 | 439.1176 | +1.7 | **4D** | **4C**–H_2_O |
|  |  | 299 | C_17_H_15_O_5_ | 299.0932 | 299.0914 | +1.8 | **4J** | **4B**–C_10_H_8_O_3_ |
|  |  | 281 | C_17_H_13_O_4_ | 281.0838 | 281.0808 | +3.0 | **4K** | **4J**–H_2_O |
|  |  | 253 | C_16_H_13_O_3_ | 253.0884 | 253.0859 | +2.5 | **4L** | **4K**–CO |
| MS^3^ | 475 | 457 | C_27_H_21_O_7_ | 457.1319 | 457.1282 | +3.7 | **4C** | **4B**–H_2_O |
|  |  | 439 | C_27_H_19_O_6_ | 439.1205 | 439.1176 | +2.9 | **4D** | **4C**–H_2_O |
| MS^4^ | 457 | 439 | C_27_H_19_O_6_ | 439.1184 | 439.1176 | +0.8 | **4D** | **4C**–H_2_O |
|  |  | 421 | C_27_H_17_O_5_ | 421.1065 | 421.1071 | –0.6 | **4E** | **4D**–H_2_O |
|  |  | 411 | C_26_H_19_O_5_ | 411.1201 | 411.1227 | –2.6 | **4F** | **4D**–CO |
|  |  | 393 | C_26_H_17_O_4_ | 393.1138 | 393.1121 | +1.7 | **4G** | **4E**–CO |
|  |  | 349 | C_25_H_17_O_2_ | 349.1255 | 349.1223 | +3.2 | **4H** | **4G**–CO_2_ |
|  |  | 321 | C_24_H_17_O | 321.1294 | 321.1274 | +2.0 | **4I** | **4H**–CO |
|  |  | 175 | C_10_H_7_O_3_ | 175.0431 | 175.0390 | +4.1 | **4M** | **4C**–C_17_H_14_O_4_ |
|  |  | 161 | C_10_H_9_O2_3_ | 161.0631 | 161.0597 | +3.4 | **4N** | **4C**–C_17_H_12_O_5_ |
| (–) MS | (518) | 517 | C_29_H_25_O_9_ | 517.1511 | 517.1504 | +0.7 | **4a** | [M–H]^–^ |
| MS^2^ | 517 | 499 | C_29_H_23_O_8_ | 499.1379 | 499.1398 | –1.9 | **4b** | **4a**–H_2_O |
|  |  | 489 | C_28_H_25_O_8_ | 489.1551 | 489.1555 | –0.4 | **4c** | **4a**–CO |
|  |  | 473 | C_27_H_21_O_8_ | 473.1256 | 473.1242 | +1.4 | **4d** | **4a**–C_2_H_4_O |
|  |  | 427 | C_26_H_19_O_6_ | 427.1176 | 427.1187 | –1.1 | **4e** | **4d**–CH_2_O_2_ |
|  |  | 383 | C_25_H_19_O_4_ | 383.1277 | 383.1289 | –1.2 | **4f** | **4e**–CO_2_ |
|  |  | 341 | C_19_H_17_O_6_ | 341.1038 | 341.1031 | +0.7 | **4g** | **4c**–C_9_H_8_O_2_ |
|  |  | 339 | C_24_H_19_O_2_ | 339.1382 | 339.1391 | –0.9 | **4h** | **4f**–CO_2_ |
|  |  | 297 | C_18_H_17_O_4_ | 297.1125 | 297.1132 | –0.7 | **4i** | **4g**–CO_2_ |
|  |  | 297 | C_17_H_13_O_5_ | 297.0803 | 297.0768 | +3.5 | **4i′** | **4g**–C_2_H_4_O |
|  |  | 268 | C_16_H_12_O_4_ | 268.0740 | 268.0741 | –0.1 | **4j** | **3d**–C_11_H_9_O_4_ |
|  |  | 253 | C_16_H_13_O_3_ | 253.0870 | 253.0870 | 0.0 | **4l** | **4i′**–CO_2_ |
|  |  | 209 | C_15_H_13_O | 209.0967 | 209.0972 | –0.5 | **4o** | **4l**–CO_2_ |
|  |  | 191 | C_10_H_7_O_4_ | 191.0360 | 191.0350 | +1.0 | **4p** | **4a**–C_19_H_18_O_5_ |
| MS^3^ | 473 | 427 | C_26_H_19_O_6_ | 427.1149 | 427.1187 | –3.8 | **4e** | **4d**–CH_2_O_2_ |
|  |  | 268 | C_16_H_12_O_4_ | 268.0701 | 268.0741 | –4.0 | **4j** | **4d**–C_11_H_9_O_4_ |
|  |  | 267 | C_16_H_11_O_4_ | 267.0648 | 267.0663 | −1.5 | **4k** | **4j**–H |
|  |  | 239 | C_15_H_11_O_3_ | 239.0750 | 239.0714 | +3.6 | **4m** | **4k**–CO |
|  |  | 211 | C_14_H_11_O_2_ | 211.0719 | 211.0765 | –4.6 | **4n** | **4m**–CO |
